# Mass Spectrometric Assessment of the Reactivity and Target Sites of 3‐Aminopropanal and 3‐Aminopropanal‐Released Acrolein in Peptides and Proteins

**DOI:** 10.1002/jms.5181

**Published:** 2025-09-26

**Authors:** Michaela Rašková, Vlastimil Dorčák, Jan Vacek, Marek Šebela

**Affiliations:** ^1^ Department of Biochemistry, Faculty of Science Palacký University Olomouc Czech Republic; ^2^ Department of Medical Chemistry and Biochemistry, Faculty of Medicine and Dentistry Palacký University Olomouc Czech Republic

**Keywords:** 3‐aminopropanal, acrolein, Michael adduct, modification, Schiff base

## Abstract

Living cells are frequently exposed to aldehydes, as these compounds are produced during metabolism, found in natural dietary sources, and present as contaminants, drugs, and pollutants. For instance, acrolein is well‐known as a toxic pollutant, but is also produced in the metabolism of polyamines, threonine, and polyunsaturated fatty acids. Another aldehyde, 3‐aminopropanal, is a byproduct of polyamine oxidation, and its cytotoxicity has been implicated in various diseases, especially those involving oxidative stress and cellular damage. 3‐Aminopropanal can readily convert to acrolein through ammonia elimination. Our objective was to compare the reactivity of these two compounds toward biomolecules. Amino acids such as cysteine and lysine, along with model peptides and proteins, were reacted with an excess of each compound. The reacted molecules were analyzed by MALDI‐TOF mass spectrometry to assess the extent of modification by examining the difference in molecular mass. Modified peptides, including those obtained by enzymatic digestion of the reacted model proteins, were subjected to tandem mass spectrometry to identify modification sites and determine the structure of the modified amino acids. The most characteristic modifications were Michael addition to cysteine and Schiff base formation with lysine, consistent with known acrolein‐induced protein modifications. Compared to acrolein, 3‐aminopropanal exhibited substantially reduced reactivity, though it generally targeted the same sites. These results represent the first experimental characterization of 3‐aminopropanal‐induced protein modifications at the molecular level, and support the notion that 3‐aminopropanal is converted to acrolein, which acts as the modifying agent.

**Abbreviations**
ACRO: acrolein
ACTH: adrenocorticotropic hormone
AGEs and ALEs: advanced glycation and lipoxidation end products
AGII: angiotensin II
APAL: 3‐aminopropanal
CHCA: α‐cyano‐4‐hydroxycinnamic acid
CPS: chronopotentiometric stripping
CTAB: cetrimonium bromide
CyA: cysteamine
DTNB: Ellman's reagent i.e. 5,5ʾ‐dithio‐bis‐(2‐nitrobenzoic acid)
DTT: dithiothreitol
FACRO: 3‐(2‐furyl)acrolein
FDP‐Lys: *N* ^ε^‐(3‐formyl‐3,4‐dehydropiperidino)lysine
FeA: ferulic acid
LIFT: low‐energy ion fragmentation technology
MP‐Lys: *N* ^ε^‐(3‐methylpyridinium)lysine
NAcCys: *N*‐acetyl‐L‐cysteine
nBSA: native (non‐reduced) bovine serum albumin
rBSA: reduced bovine serum albumin
SA: sinapinic acid
SST: somatostatin
TFA: trifluoroacetic acid
UBQ: ubiquitin
YADH: yeast alcohol dehydrogenase

## 1 Introduction

**FIGURE 1:**
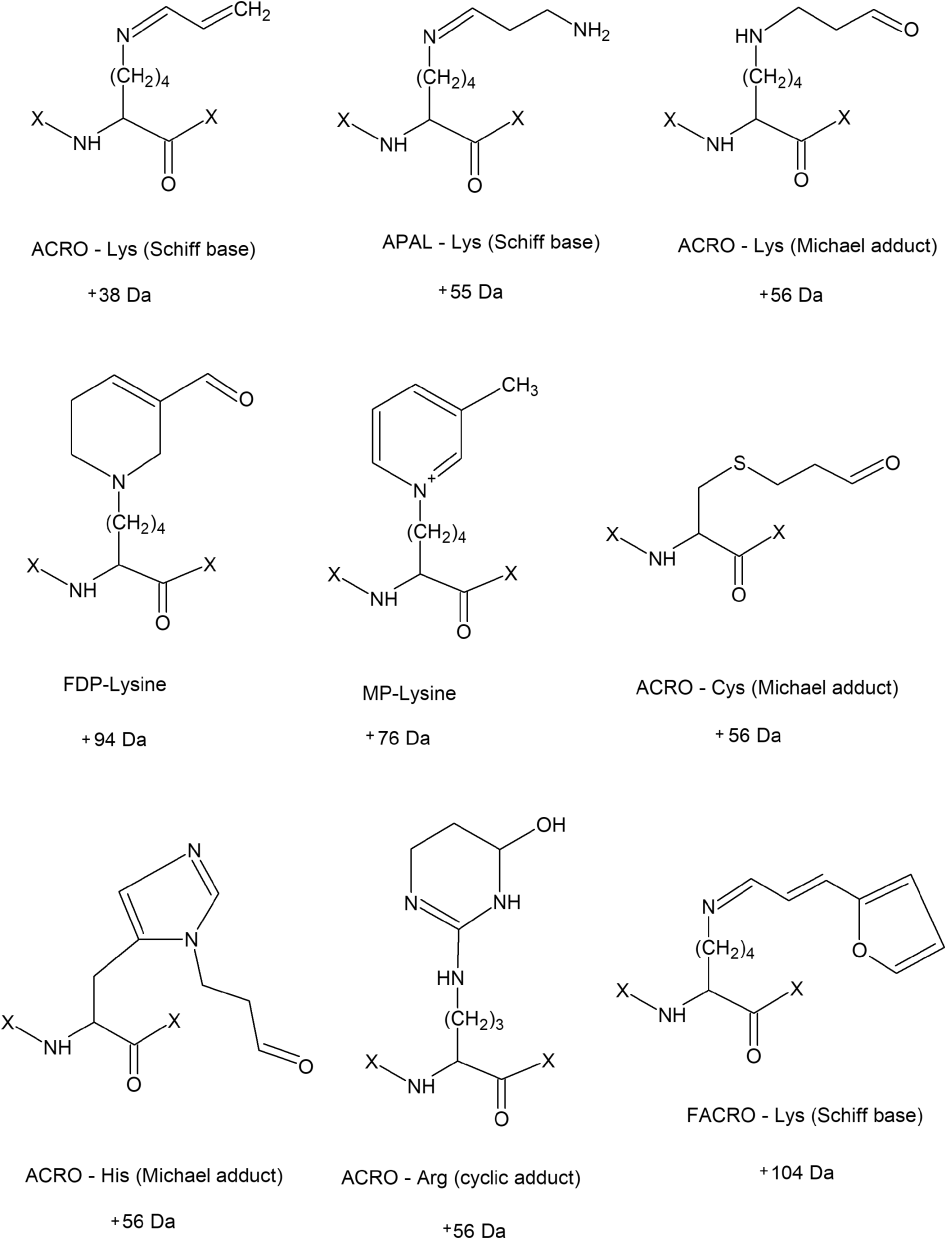
Modification of amino acid residues by ACRO and APAL. The scheme includes Schiff bases of Lys (for both ACRO and APAL), Michael adducts of Lys, Cys, and His (for ACRO), a cyclic adduct of Arg (for ACRO), as well as *N* ^ε^‐(3‐formyl‐3,4‐dehydropiperidino)lysine (FDP‐Lys), and *N* ^ε^‐(3‐methylpyridinium)lysine (MP‐Lys), both for ACRO. In addition, a Schiff base of Lys reacted with 3‐(2‐furyl)acrolein (FACRO) is depicted as the reagent was used in this study for a comparison. The corresponding molecular mass increases upon modifications are provided too.

Reactive aldehydes are a diverse class of electrophilic compounds that are produced endogenously as byproducts of lipid peroxidation, sugar metabolism, and the oxidative degradation of polyamines and amino acids. These reactive aldehydes are also introduced into the body through environmental pollutants, food processing, and drugs [1, 2, 3]. Among them, short‐chain aldehydes such as acrolein (ACRO), methylglyoxal, and crotonaldehyde have attracted considerable attention due to their high reactivity with proteins, leading to significant biological consequences [1]. Polyamines, such as spermine and spermidine (along with their precursor putrescine), are essential for cellular growth and development. However, the oxidative catabolism of these compounds by copper amine oxidases and flavoprotein polyamine oxidases generates cytotoxic aminoaldehydes, including 3‐aminopropanal (APAL). These aminoaldehydes can further decompose to release ACRO, a highly reactive α,β‐unsaturated aldehyde, which is known for its ability to covalently modify proteins through Michael‐type additions or Schiff base formations with nucleophilic residues such as cysteine, lysine, and histidine [1, 4, 5, 6] (Figure 1). These modifications can result in protein carbonylation, cross‐linking, and the formation of advanced glycation and lipoxidation end products (AGEs and ALEs), which are implicated in various oxidative stress‐related diseases, including diabetes, atherosclerosis, and neurodegenerative disorders [7].

ACRO, in particular, has been extensively studied due to its high cytotoxicity and its role in promoting inflammation‐related diseases such as Alzheimer's disease and atherosclerosis [8, 9, 10]. This aldehyde is not only produced endogenously, but also introduced through environmental exposure, such as cigarette smoke and the combustion of fuels [11]. Its reactivity stems from its ability to form covalent adducts with protein residues, thereby altering protein function and contributing to cellular damage. ACRO forms Schiff bases and Michael adducts with proteins, which can further react to form cyclic or cross‐linked products, exacerbating its cytotoxic effects [6, 12, 13]. APAL exerts cytotoxicity by accumulating in lysosomes, causing their rupture and subsequent release of lysosomal enzymes. This process is accompanied by apoptosis or necrosis, leading to damage in pathologic tissues, such as in ischemic brain [14]. The role of protein modification by reactive aldehydes is of particular interest in the context of disease. The accumulation of AGEs and ALEs in proteins can disrupt their structure and function, potentially contributing to the progression of conditions such as chronic kidney disease, cardiovascular diseases, and neurodegeneration [7]. For example, the carbonylation of proteins, often used as a biomarker for oxidative stress, is commonly observed in disease states where reactive aldehydes play a significant role [15].

Recent advances in analytical techniques, such as mass spectrometry (MS) and immunochemical methods, have allowed for the detection and characterization of aldehyde‐induced protein modifications [16, 17]. These methods provide crucial insights into the structural changes in proteins after aldehyde exposure. Moreover, understanding the specific sites and types of modifications can help to elucidate the mechanisms by which these reactive aldehydes contribute to disease pathogenesis. The aim of this work was to investigate the modification efficiency of APAL in reaction mixtures containing model amino acids (Cys and Lys), peptides (adrenocorticotropic hormone fragment 18–39, angiotensin II, somatostatin 28, and insulin), and proteins (alcohol dehydrogenase, bovine serum albumin, and ubiquitin). Although APAL has repeatedly been described as an endogenous source of ACRO, formed by ammonia elimination from its amino group, protein modifications induced by APAL have not yet been studied at the molecular level using chemical analysis. To our knowledge, the chemistry of APAL‐induced modifications, including the types of modifications and preferred target sites, has not been fully explored. Matrix‐assisted laser desorption/ionization time‐of‐flight (MALDI‐TOF) mass spectrometry (MS) was employed to elucidate the modification processes induced by APAL, allowing us to attribute these modifications to the released ACRO and to highlight differences compared to the action of ACRO alone.

## 2 Materials and Methods

### 2.1 Chemicals

Alpha‐cyano‐4‐hydroxycinnamic acid (CHCA), adrenocorticotropic hormone (ACTH) fragment 18–39, angiotensin II (AGII), sinapinic acid (SA), and somatostatin (SST) 28 were purchased from Bruker Daltonik (Bremen, Germany). *N*‐Acetyl‐L‐cysteine (NAcCys), acrolein (ACRO) diethylacetal, alcohol dehydrogenase from yeast (YADH), 3‐aminopropanal (APAL) diethylacetal (1‐amino‐3,3‐diethoxypropane), bovine serum albumin (BSA), cetrimonium bromide (CTAB), cysteamine (CyA) hydrochloride, L‐cysteine hydrochloride, DL‐dithiothreitol (DTT), Ellmanʾs reagent (DTNB), i.e., 5,5ʾ‐dithio‐bis‐(2‐nitrobenzoic acid), endoproteinase Glu‐C (V8) from *Staphylococcus aureus* , ferulic acid (FeA), *trans*‐3‐(2‐furyl)acrolein (FACRO), insulin from bovine pancreas, iodoacetamide, L‐lysine hydrochloride, 2‐mercaptoethanol, SOLu trypsin, trifluoroacetic acid (TFA), ubiquitin (UBQ) from bovine erythrocytes, and ZipTip‐C_18_ pipette tips were from Merck (Steinheim, Germany). All other chemicals were of analytical purity grade.

Free ACRO and APAL were obtained by heating the respective diethylacetals in diluted hydrochloric acid. APAL or ACRO diethylacetals were added in 10‐μl portions to 0.5 mL of 0.5 mol·l^−1^ HCl. The mixture was incubated at 100°C for 10 min and then cooled on ice. Then, it was neutralized with 25% ammonium hydroxide and adjusted with deionized water to a final concentration of free aldehyde of 60 mmol·L^−1^. The stock solution of FACRO was prepared in ethanol at a concentration of 100 mmol·L^−1^.

### 2.2 Reaction of Aldehydes With Amino Acids, Peptides, and Proteins

The amino acids under investigation (Cys, Lys, NAcCys) and CyA were dissolved in 50 mmol·L^−1^ NH_4_HCO_3_, pH 8.0, to prepare 1 mmol·L^−1^ solutions. They were incubated in the presence of 3 mmol·L^−1^ APAL, ACRO or FACRO in a thermomixer (Eppendorf, Hamburg, Germany) at 37°C and 500 RPM for 30 min before MALDI‐TOF MS analysis.

All peptides and UBQ were dissolved to obtain 100 μmol·L^−1^ solutions in 50 mmol·L^−1^ NH_4_HCO_3_, pH 8.0, with the exception of SST 28, which was prepared at 50 μmol·L^−1^. Insulin was reduced with 2‐mercaptoethanol in a molar ratio of 1:5. The aldehydes APAL and ACRO were added to the peptide solutions (10–100 μL) to a final concentration of 5 mmol·L^−1^, providing a molar reagent excess of 50:1. The reaction mixtures were incubated at 37°C and 500 RPM for 30 min to 108 h. After incubation, peptides were recovered by evaporating the solvent in a vacuum centrifuge, and were reconstituted in 10 μL of 0.1% TFA for purification using ZipTip‐C_18_ pipette tips. Subsequently, they were analyzed either by nanoflow liquid chromatography (nLC) coupled to MALDI‐TOF/TOF tandem mass spectrometry (MS/MS) on an instrument equipped with a LIFT (low‐energy ion fragmentation technology) cell, which increases the energy of fragment ions to ensure their correct entry into the second TOF analyzer for detection, or by a manual MS/MS on the same instrument. Further details are provided below. After modification, SST 28, insulin, and UBQ were subjected to overnight digestion at 37°C using peptidases (trypsin and/or Glu‐C) with the enzyme added in a weight ratio of 1:100 relative to the peptide substrate. The digests were processed by evaporating the solvent in a vacuum centrifuge, reconstituted in 10 μL of 0.1% TFA, purified using ZipTip‐C_18_ pipette tips, and transferred to vials for nLC‐MALDI‐LIFT‐TOF/TOF MS/MS analysis.

Two proteins were analyzed for their modifications induced by APAL, ACRO, and FACRO: YADH and BSA. Because of the content of 17 disulfide bonds, BSA was used not only as a native protein, but also after a reduction treatment with DTT in 50 mmol·L^−1^ NH_4_HCO_3_, pH 8.0. DTT was added in an excess of 2:1 relative to the molar amount of the disulfides in the sample. The reduced BSA (rBSA) was processed by centrifugal ultrafiltration (10‐kDa cutoff) with a repeated buffer exchange to remove the unreacted reagent. All protein samples, 100 μmol·L^−1^ solutions in 50 mmol·L^−1^ NH_4_HCO_3_, pH 8.0, were reacted with a molar excess of 50:1 of the aldehydes at 37°C and 400 RPM for 16 h. For rBSA, the excess used was 200:1. After incubation, protein samples were in part treated with a 2× Laemmli sample buffer and separated by SDS‐PAGE in 4% concentrating and 12% resolving polyacrylamide gels [18]. The modified proteins were loaded in amounts of 8 and 16 μg per lane. The resolving gels were finally stained with Coomassie Brilliant Blue G250 and then subjected to the procedure of protein band excision, reduction, alkylation, and in‐gel digestion by trypsin [19]. The digests were desalted using ZipTip‐C_18_ prior to nLC‐MALDI‐LIFT‐TOF/TOF MS/MS analysis. The residual proteins were reduced, alkylated and subjected to an in‐solution digestion by trypsin (1:20, w/w) followed by desalting of aliquots and MS/MS analysis as above.

### 2.3 Mass Spectrometric Measurements (MALDI‐TOF)

MALDI‐TOF MS was conducted in a Microflex LRF 20 instrument (Bruker Daltonik) equipped with a 60‐Hz nitrogen laser (λ_max_ = 337 nm). The instrument was set to reflector positive ion mode and operated using the spectrum acquisition software flexControl 3.4 (Bruker Daltonik). All mass spectra were evaluated using flexAnalysis 3.4 (Bruker Daltonik).

The matrix used for low‐molecular‐weight compounds (amino acids and their derivatives; in the *m/z* region of 0–2100) was CHCA, 0.1 mol·L^−1^ in acetone:water 4:1,v/v, containing 0.1 mmol·L^−1^ CTAB [20]. The parameters of the instrument were as follows: IS1 voltage of 19 kV, IS2 voltage of 16.2 kV, lens voltage of 8.9 kV, reflector voltage of 20 kV, and detector voltage of 1672 V. Delayed extraction was employed with the pulsed ion extraction time set to 150 ns. Initially, 1‐μL aliquots of the matrix were deposited at the sample positions on an MSP BigAnchor 96 bc target plate (Bruker Daltonik) and left to dry at ambient temperature. Reaction mixture samples (1 μL) were then applied onto the crystallized matrix and left to dry. Matrix peaks at *m/z* 172 ([CHCA‐H_2_O + H]^+^), 190 ([CHCA+H]^+^), 284 (cetrimonium, i.e., [CTAB − Br]^+^), and 379 ([2CHCA + H]^+^) were used for internal calibration. Mass spectra were accumulated from 1000 single‐pulse shots.

For the MALDI‐TOF MS of peptides, the parameters of the instrument were the same as above, except for selecting a peptide mass region of *m/z* 500–4500 and adjusting the detector voltage to 1707 V. The external calibration for peptides was achieved using the Peptide Calibration standard II (Bruker Daltonik) containing nine standard peptides (signals between *m/z* 757 and 3147). The matrix solution used contained CHCA, 7 mg·mL^−1^ in acetonitrile:2.5% TFA, 7:3, v/v. Peptide samples (0.5 μL) were deposited onto the MSP BigAnchor 96 bc target plate, overlaid immediately with the matrix solution (0.5 μL), and left to dry in air at ambient temperature for crystallization. Mass spectra were accumulated from 2000 laser shots.

Intact UBQ and its modified forms were measured using FeA:SA as a matrix (5:15 mg·mL^−1^ in acetonitrile:2.5% TFA, 7:3, v/v). The instrument was calibrated externally using Protein Calibration Standard I (Bruker Daltonik) containing several proteins (signals between *m/z* 5734 and 16 952), and the inspected mass range was set to *m/z* 2000–20 000. The parameters of the instrument in the reflector positive ion mode were as follows: IS1 voltage of 19 kV, IS2 voltage of 15.5 kV, lens voltage of 9.0 kV, reflector voltage of 20 kV, and detector voltage of 1680 V. Delayed extraction was employed with the pulsed ion extraction time set to 500 ns. UBQ samples (1 μL) were deposited onto the MSP BigAnchor 96 bc target plate, overlaid immediately with the matrix solution (1 μL), and left to dry in air at ambient temperature for crystallization. Mass spectra were accumulated from 2000 laser shots.

### 2.4 MALDI‐LIFT‐TOF/TOF MS/MS

Peptide samples after the digestion of model proteins and larger peptides were purified from the reaction mixtures using ZipTip‐C_18_ pipette tips according to the manufacturer's instructions. The purified peptides were reconstituted in 10 μL of 0.1% TFA and subjected to a chromatographic purification by nLC connected offline to MALDI‐LIFT‐TOF/TOF MS/MS performed in an ultrafleXtreme mass spectrometer (Bruker Daltonik). The instrument was equipped with a 2‐kHz smartbeam‐II laser (λ_max_ = 355 nm). All experimental details for the automated chromatography of peptides, collecting fractions during the gradient elution, spotting them with CHCA matrix on the target plate, and MALDI measurements have already been described [21, 22]. For manual MS/MS analyses of modified peptides, the parameters of the instrument (a LIFT method was used) were as follows: IS1 voltage of 7.5 kV, IS2 voltage of 6.8 kV, lens voltage of 3.5 kV, reflector voltage of 29.5 kV, reflector 2 voltage of 14.15 kV, LIFT1 voltage of 19 kV, LIFT2 voltage of 3.15 kV, detector voltage of 2476 V, delayed extraction with a pulsed ion extraction time of 50 ns, and precursor ion selection window range of 8 Da. Precursors for collision‐induced dissociation with nitrogen gas were selected manually in flexAnalysis 3.4. Precursor spectra were accumulated from 10 000 laser shots, and fragment spectra from 20 000–40 000 shots in flexControl 3.4. Raw data files were processed for database searches (a custom database with all necessary sequences was used) using ProteinScape 3.1 (Bruker Daltonik) or Peaks X (Bioinformatic Solutions, ON, Canada) including variable modifications with (1) ACRO: Michael adducts (Cys, Lys, Arg, and His); Schiff base (Lys); FDP‐Lys, i.e., *N* ^ε^‐(3‐formyl‐3,4‐dehydropiperidino)lysine; MP‐Lys, i.e., *N* ^ε^‐(3‐methylpyridinium)lysine; and (2) APAL: Schiff base (Lys).

### 2.5 Spectrophotometry

The content of free cysteine SH‐groups in proteins and modified proteins was determined by a spectrophotometric assay using DTNB [23, 24]. The reaction was carried out in 50 mmol·L^−1^ Tris–HCl, pH 8.0, containing 0.1 mol·L^−1^ KCl and 1 mmol·L^−1^ ethylenediaminetetraacetic acid. The mixture, containing DTNB (1 mmol·L^−1^) and the protein sample (2–20 μmol·L^−1^) at a concentration ratio of at least 50:1, was incubated at 37°C for 30 min. Absorption at 412 nm (ε_412_ = 14 150 mol^−1^·L·cm^−1^) was then read against a blank composed of the buffer and DTNB, the protein solution being replaced with water.

### 2.6 Electrochemistry

Cysteine (60 and 6 mmol·L^−1^) was treated with free APAL or ACRO (60 mmol·L^−1^; after hydrolysis of the corresponding acetals, see the Chemicals section) in 0.15 mol·L^−1^ Na‐phosphate buffer, pH 8.0, for 1 h. Chronopotentiometric stripping (CPS) analysis was conducted after the treatment with the aldehydes in 1:0, 1:1, and 1:10 molar ratios and a 10 μmol·L^−1^ final concentration of Cys in the cell. Electrochemical measurements were carried out using a μAutolab III analyzer (EcoChemie) connected to a VA‐stand 663 (Metrohm) in a three‐electrode setup, comprising a hanging mercury drop electrode (HMDE) as the working electrode, an Ag|AgCl|3 M KCl as the reference electrode, and a glassy carbon rod as the auxiliary electrode. Solutions were deaerated with argon prior to measurement. Conventional CPS analysis with HMDE employed a 60 s accumulation time at an accumulation potential *E*_A_ of 0.1 or −0.25 V. Chronopotentiograms were recorded at a stripping current intensity of −5 μA from an initial potential equal to *E*_A_.

## 3 Results

### 3.1 Modifications of Amino Acids

Initial experiments were conducted to evaluate the results of chemical modifications induced in free amino acids (Cys, Lys, NAcCys) and CyA by APAL, ACRO, and FACRO. Ammonium bicarbonate was chosen as the reaction buffer because it is MS‐compatible and its slightly basic pH is more favorable for amino group reactions compared to the neutral pH inside cells. Control mass spectra of free APAL in ammonium bicarbonate contained an [M + H]^+^ peak at *m/z* 74 and two peak pairs with a mass difference of 55 Da: (1) *m/z* 111 and 166; (2) *m/z* 128 and 183, indicating the presence of possible APAL adducts: 111 = (2 × 73–2 × 18) plus protonation, 166 = (3 × 73–3 × 18) plus protonation, 128 = (2 × 73–2 × 18) plus ammonium, 183 = (3 × 73–3 × 18) plus ammonium.

**FIGURE 2:**
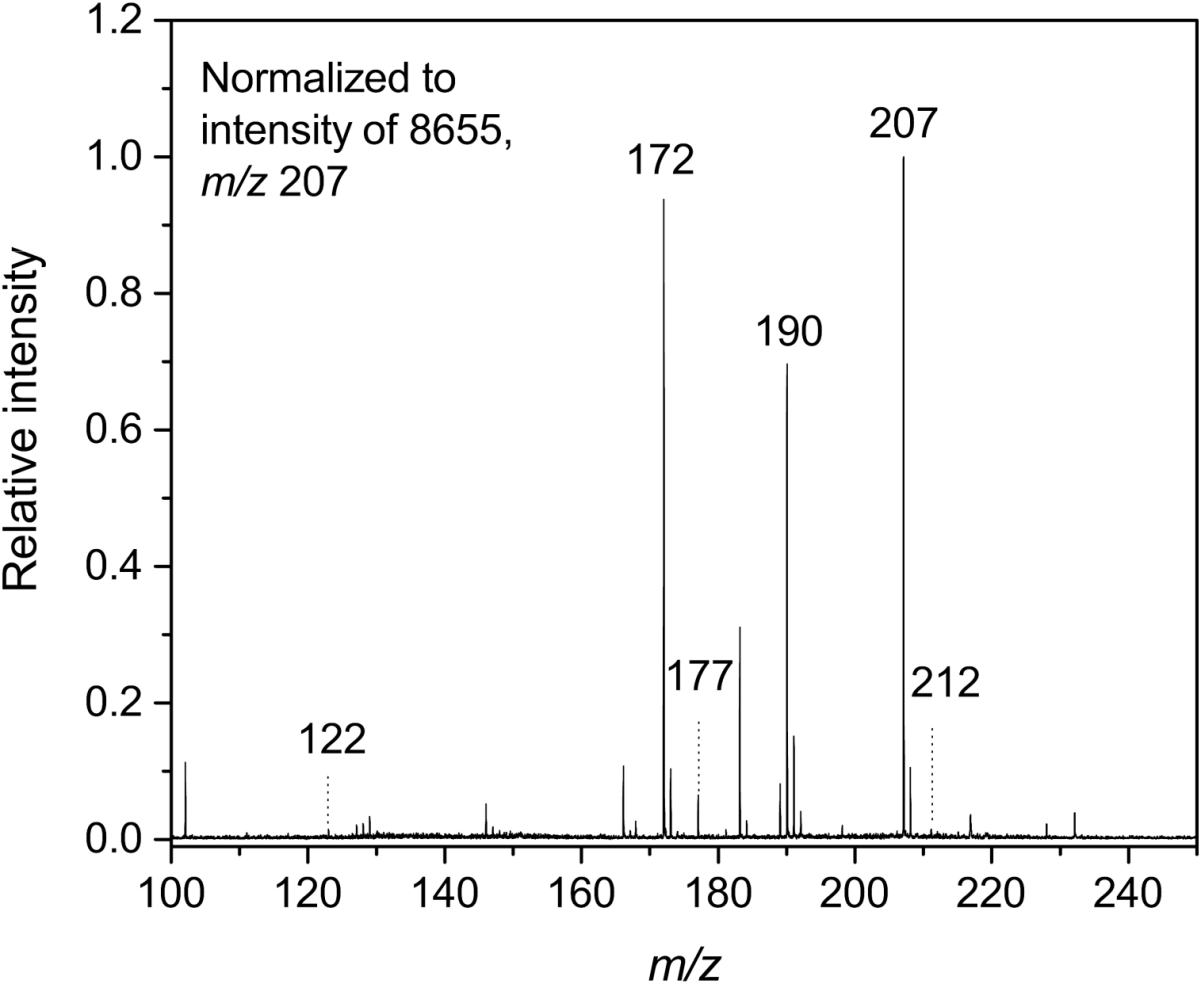
MALDI‐TOF mass spectrum of a reaction mixture containing Cys and APAL. The molar ratio of the compounds was 1:3, and the reaction occurred in 50 mmol·L^−1^ NH_4_HCO_3_ at pH 8 and 37°C. The signal at *m/z* 177 corresponds to a product of the Cys reaction with APAL, while residual Cys appears at *m/z* 122. The CHCA matrix gave distinguishable peaks at *m/z* 172 ([CHCA‐H_2_O + H]^+^), 190 ([CHCA+H]^+^), 207 ([CHCA+NH_4_]^+^), and 212 ([CHCA+Na]^+^).

The starting compounds gave clear [M + H]^+^ peaks in MALDI‐TOF mass spectra at *m/z* 122 (Cys), 164 (NAcCys), 147 (Lys), and 78 (CyA). The reactions with APAL resulted in product peaks at *m/z* 177 (modified Cys), 237 (modified NAcCys), 202 (modified Lys), and 133 (modified CyA). Figure 2 shows the result of cysteine modification. The ACRO‐induced modifications were observed by the presence of the following peaks: *m/z* 160 and 178 (modified Cys), 220 (modified NAcCys), 185 and 241 (modified Lys), and 116 (modified CyA). With the use of FACRO, chosen as a control expected to not form Michael adducts under the given conditions (Figure 1), modification product signals were observed at *m/z* 226 (modified Cys), 251 (modified Lys), and 182 (modified CyA). No modification product was observed in the reaction mixture of NAcCys. Mass spectra of free APAL showed its [M + H]^+^ peak at *m/z* 74, free ACRO and FACRO could not be detected.

### 3.2 Modifications of Peptides

Four model peptides (ACTH 18‐39, AGII, insulin, and SST 28) and a small protein (UBQ) were subjected to reactions with ACRO and APAL. The reaction mixtures were then analyzed by MALDI‐TOF MS and MALDI‐LIFT‐TOF/TOF MS/MS (optionally also coupled to nLC peptide separation when the modified insulin and UBQ were additionally digested by Glu‐C and/or trypsin, and SST 28 by trypsin) to detect induced modifications. Table 1 summarizes the obtained results, which confirmed that APAL‐released ACRO and ACRO itself modified largely overlapping target sites, and a major difference was in the extent and yield of the modification, represented by the intensity of the MS peaks.

**TABLE 1:** Peptide modifications induced by APAL and ACRO.

Peptide or protein	Sequence
Bovine insulin	GIVEQCCASV CSLYQLENYC N (chain A) FVNQHL **C** GSH LVEALYLVCG ERGFFYTP **K** A (chain B)
Human SST 28	**S** ANSNPAMAP RERKAGCKNF FWKTFTSC
Human ACTH 18‐39	**R** PVKVYPNGA EDESAEAFPL EF
Human AGII	**D** RVYI**H**PF
Bovine UBQ	**M** QIFV **K** TLTG **K** TITLEVEPS DTIENV **K** AKI QD **K** EGIPPDQ QRLIFAG **K** QL EDG**R**TLSDYN IQ **K** ESTL**H**LV LRLRGG

*Note:* Ubiquitin is also included, although it is considered a small protein rather than a peptide. The presence of the modifications at specific sites was demonstrated using MALDI‐LIFT‐TOF/TOF MS/MS. Modifications confirmed after the reaction with ACRO are in bold; modifications confirmed after the reaction with APAL are underlined.

**FIGURE 3:**
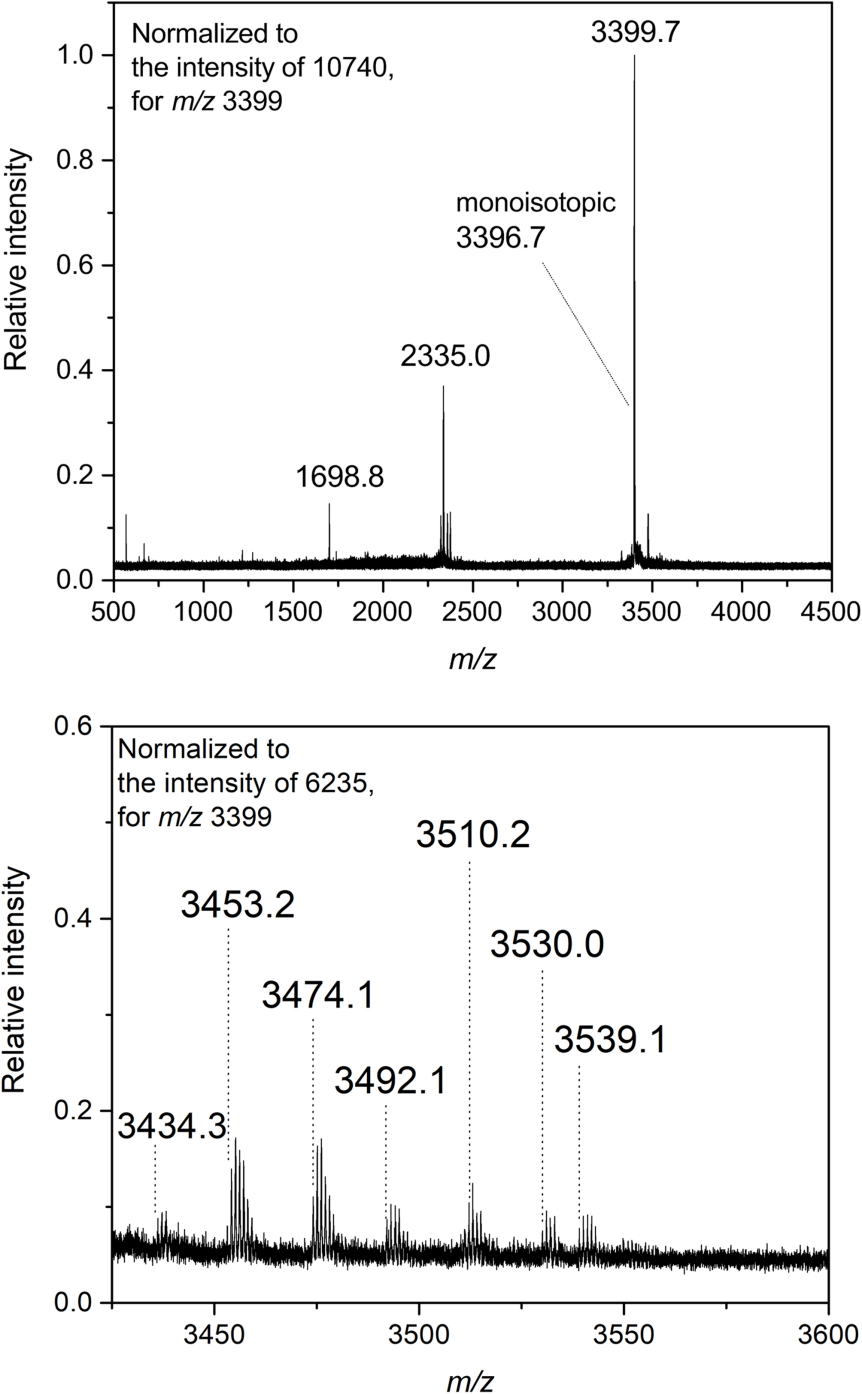
MALDI‐TOF mass spectra of modified insulin. The top panel shows signals of two separate and unmodified insulin chains: chain A (*m/z* 2335.0), and chain B (*m/z* 1698.8 and 3396.7). The bottom panel shows a magnified region with signals of the ACRO‐modified B‐chain (incubation for 5 h) at *m/z* 3453.2, 3492.1, 3510.2, and 3530.0. The other visible signals (*m/z* 3434.3, 3474.1, and 3539.1) were already present in the control spectrum, and thus do not correspond to the studied chemical modification.

The formation of Michael adducts, Schiff bases, FDP‐Lys, and MP‐Lys was confirmed, with the first two being the most frequent. Figure 3 shows MALDI‐TOF mass spectra of bovine insulin before and after the modification with ACRO. After its initial reduction to break down the disulfide bonds and release the chains A and B, three dominant signals were observed at *m/z* 1699, 2335, and 3397 (Figure 3), which were attributed to [M_B_ + 2H]^2+^, [M_A_ + H]^+^, and [M_B_ + H]^+^ pseudomolecular ions of insulin chains, respectively, in accordance with a previous report [25]. The modified B‐chain provided additional signals such as those at *m/z* 3453.2, 3492.1, 3510.2, and 3530.0, which were well recognizable after the reaction with ACRO or after adding APAL as the reagent (Figure 3). The mass differences suggested the presence of a Michael adduct with an ACRO moiety (+56), Schiff base (+38), FDP‐Lys (+94), MP‐Lys (+76), or some combinations of the above. The added mass of +95 Da in the peak at *m/z* 3492 does not appear to correspond to a specific known modification. It may represent a combination of two modifications, such as a +56 Da Michael addition and an additional +38 Da mass shift. The ~1 Da discrepancy could result from calibration drift, as the nearest calibration point was at *m/z* 3147, or from a minor modification such as deamidation (e.g., Q → E or N → D). Similarly, the added mass of +133 Da observed at *m/z* 3530 may indicate a combination of modifications, e.g., two +38 Da shifts and a +56 Da Michael addition, 38 + 94 Da, or 56 + 76 Da. The A chain was also modified, as deduced from the newly appeared peaks at *m/z* 2411.0, 2429.1, 2467.1, and 2487.0. The MS/MS results confirmed several of the existing modifications of the B‐chain after its cleavage into smaller peptides (e.g., *m/z* 1538.7 and 1162.6) by Glu‐C: the Michael adduct at C7 (for both ACRO and APAL) and MP‐Lys at K29 (for ACRO) elucidating the mass shifts observed with the intact B‐chain. The formation of ACRO adducts was much faster than of their counterparts with APAL‐derived ACRO. The intensity ratio of the peaks at *m/z* 3492 (modified insulin) and 3397 (unmodified insulin) was 0.22 after the incubation with ACRO for 5 h. There was no recognizable peak at *m/z* 3492 with APAL after the same incubation period, but then it reached a value of 0.39 after 48 h.

Only N‐terminal amino group modifications were confirmed by MS/MS for the reacted ACTH 18‐39 and SST 28. The modifications of ACTH with APAL and ACRO included a Schiff base (*m/z* 2503.2), and a Michael adduct (*m/z* 2521.2). Additionally, two other N‐terminal modifications with ACRO were inferred from the MS/MS spectra (FDP‐like, *m/z* 2559.3, and MP‐like, *m/z* 2541.2). The formation of such modifications at the N‐termini can occur in the presence of ACRO and prolonged incubations [13]. Similarly, a Schiff base (*m/z* 1153.5), a Michael adduct (*m/z* 1171.5 and 1187.5, with the latter peptide also showing methionine oxidation), and a possible FDP‐derivative of the N‐terminal amino group (*m/z* 1209.6 and 1225.6) were confirmed in SST 28 reacted with ACRO. In contrast, only a Michael adduct (*m/z* 1187.5) was observed when using APAL as the reagent. Two modifications of the N‐terminal amino group of AGII were found after the reaction with APAL: a Schiff base (*m/z* 1084.6) and a Michael adduct (*m/z* 1102.6). When ACRO was used for the treatment, an additional FDP‐like modification was inferred from the MS/MS spectra (*m/z* 1140.6), plus a combination of FDP at the N‐terminus and a Michael adduct at H6 (*m/z* 1196.6).

**FIGURE 4:**
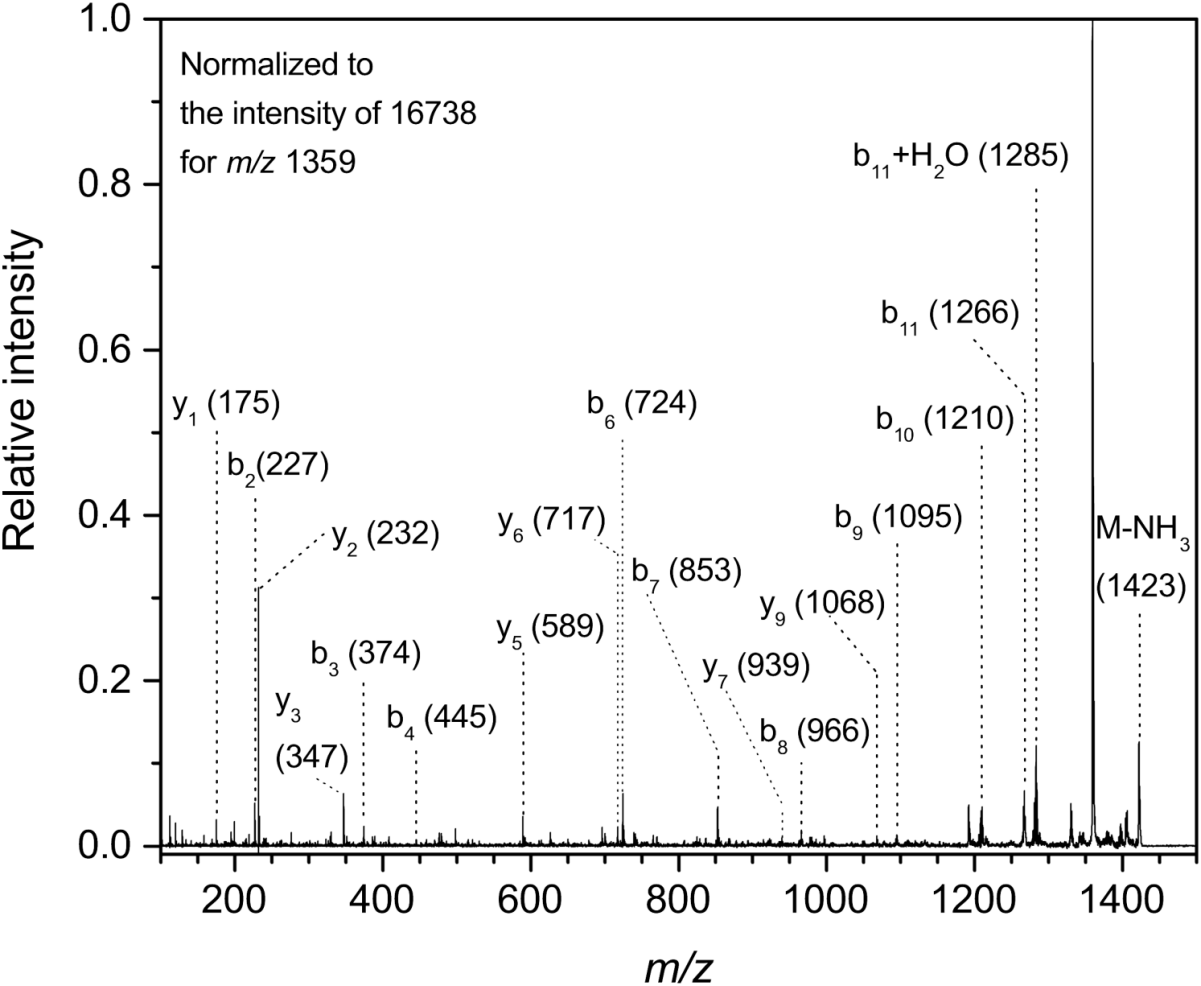
MS/MS spectrum of a tryptic peptide (*m/z* 1440.8) from modified ubiquitin. This precursor peptide was detected in tryptic digests of ubiquitin, modified either by ACRO (as shown here) or APAL. The spectrum was acquired using an ultrafleXtreme MALDI‐LIFT‐TOF/TOF instrument after nLC separation of the tryptic digest. The fragmentation pattern corresponds to the sequence LIFAGK*QLEDGR, in which the asterisk indicates the presence of an ACRO‐induced modification, specifically FDP‐Lys (+94.04).

Intact mass determination of the reacted UBQ indicated a heterogeneity of modified forms. Unmodified protein gave a characteristic sharp peak of the monomer (*m/z* 8564.9). After incubation with ACRO for 2 h, newly formed signals were registered at *m/z* 8603, 8621, 8641, 8659, 8677, 8697, 8717, 8735, and other masses up to *m/z* ~ 9000. The presence of modified UBQ was also obvious when measuring with the reaction mixture containing APAL, but after 48 h of incubation the intensity was still very low, except for *m/z* 8603 and 8621. This indicated similar target sites for the modification and generation of APAL‐derived ACRO. The modified UBQ was digested by Glu‐C and trypsin to generate modified peptides analyzable by nLC‐MALDI‐LIFT TOF/TOF MS/MS. The sequenced peptide precursors were between *m/z* 1594.8 and 2000.4 (Glu‐C), or *m/z* 1123.7–2364.3 (trypsin). Modifications in UBQ were confirmed according to expectations at Lys residues, since there is no Cys present in the sequence. After the reaction with ACRO, these included Michael adducts (K33, K48), Schiff bases (K33, K48), FDP‐Lys (K6, K33, K48), and MP‐Lys (K6, K11, K27, K33, K48, and K63). Michael adducts were additionally found at the N‐terminal amino group (M1), H68 and R54. Figure 4 shows the confirmation of the modification in a tryptic peptide with *m/z* 1440.8. The deduced sequence LIFAGK*QLEDGR is in accordance with the presence of an FDP moiety at K48. The amount of ACRO‐related modifications, which were confirmed in the reaction mixture with APAL, was lower. The major modification was again MP‐Lys (K6, K11, K27, K33, K48, K63), but Schiff bases were also found (K11, K48), as well as a Michael adduct (H68) and an FDP‐Lys (K48).

### 3.3 Modifications of Proteins

Two proteins larger in size than UBQ ‐ YADH and BSA ‐ were reacted with APAL and ACRO, digested by trypsin, and analyzed at the peptide level using nLC‐MALDI‐LIFT‐TOF/TOF MS/MS. Because the Cys residues in BSA (except for C34) are present in the form of disulfides, the protein was reduced by DTT, and studied in both the native (nBSA) and reduced (rBSA) form. As can be seen in Table 2, the lysine residues K28, K30, K81, K224, K226, K332, and K335 in yeast ADH were modified by APAL, ACRO, and FACRO, yielding Schiff bases. C278 reacted with APAL and ACRO, yielding a Michael adduct. FDP‐Lys (K207) was only found when using ACRO.

**TABLE 2:** Modifications induced by APAL‐derived ACRO, ACRO and FACRO in yeast ADH.

*m/z*	Modification type	Modified residue(s)
Modification of YADH by APAL‐derived ACRO		
969.6	2 × ACRO–Schiff	**K28, K30**
1192.6	ACRO–Michael	**H16**
1354.6	CAM, ACRO–Michael	C277, C278
1645.9	2 × ACRO–Schiff	**K224, K226**
1656.8	ACRO–Schiff	**K207**
1912.0	2 × ACRO–Schiff	**K332, K335**
2057.0	ACRO–Schiff	**K81**
Modification of YADH by ACRO		
931.5	ACRO–Schiff	K28
969.6	2 × ACRO–Schiff	**K28, K30**
1354.6	CAM, ACRO–Michael	C277, C278
1645.8	2 × ACRO–Schiff	**K224, K226**
1712.9	FDP	**K207**
1912.0	2 × ACRO–Schiff	**K332, K335**
1928.0	2 × ACRO–Schiff, Oxidation	**K332, K335**, **M333**
1964.1	2 × ACRO–Schiff	**K28, K30**
2057.0	ACRO–Schiff	**K81**
2466.3	2 × ACRO–Schiff	**K81, K84**
Modification of YADH by FACRO		
1101.6	2 × FACRO–Schiff	**K28, K30**
1777.9	2 × FACRO–Schiff	**K224, K226**
2044.0	2 × FACRO–Schiff	**K332, K335**
2598.3	2 × FACRO–Schiff	**K81, K84**

*Note:* All these modifications were confirmed by nLC‐MALDI‐LIFT‐TOF/TOF MS/MS of tryptic peptides after reaction at pH 8.0 and 37°C for 16 h. The abbreviation CAM stands for carbamidomethyl cysteine, Schiff stands for a Schiff base, Michael for a Michael adduct, Oxidation for a Met oxidation, and FDP for *N* ^ε^‐(3‐formyl‐3,4‐dehydropiperidino)lysine. Underlined text indicates identifications from in‐gel digestion, while bold text indicates results from in‐solution digestion. Numbering corresponds to the sequence in the UniProt database (Accession no. ADH1_YEAST), including N‐terminal methionine.

The observed modifications of nBSA and rBSA with APAL or ACRO are summarized in Table 3. They mostly included Schiff bases of Lys and Michael adducts of Cys due to binding ACRO moieties. The former modifications were typically found to occur after in‐solution digestion, while the latter were detected primarily after in‐gel digestion. Michael adducts were also detected at K587 and R122. The modifications at K587, C500, and C582 were always present, regardless of the reagent used and protein redox status. APAL modifications without ammonia elimination were found at K130 and K587. The MS/MS analysis of rBSA modified by FACRO (no Cys modification was expected) resulted in the identification of Schiff bases at K256, K263, K266, K495, K498, K557, K559, and K561 after the in‐solution digestion. No FACRO‐induced modification was detected after the treated nBSA had been digested in‐gel. In addition to ACRO‐containing Schiff bases (+38 Da), we were also able to confirm APAL‐containing Schiff bases (+55 Da), but as a small proportion of all modifications.

**TABLE 3:** Modifications induced by APAL and ACRO in BSA.

Native BSA—modifications by APAL/APAL‐derived ACRO			
Type of modification	Modified Lys residues	Modified Cys residues	Other modified residues
Schiff base	K130*, **K386**, **K544**, **K547**, K587*	N.A.	N.A.
Michael adduct	K587	C114, C115, C471, **C500** , **C582** , C590	R122
Reduced BSA—modifications by APAL/APAL‐derived ACRO			
Type of modification	Type of modification	Type of modification	Type of modification
Schiff base	**K36**, K130*, **K140**, K463, K587*	N.A.	N.A.
Michael adduct	K587	**C125**, **C288**, **C312**, C392, **C471**, C500, C582, C590	None
Native BSA—modifications by ACRO			
Type of modification	Type of modification	Type of modification	Type of modification
Schiff base	**K138**, **K140**, **K263**, **K266**, **K412**, **K420**, **K544**, **K547**	N.A.	N.A.
Michael adduct	K587	**C268**, C269, C415, **C500** , **C582** , C590	**R122**
Reduced BSA—modifications by ACRO			
Type of modification	Type of modification	Type of modification	Type of modification
Schiff base	**K36**, **K256**, **K263**, **K266**	N.A.	N.A.
Michael adduct	K587	C125, C268, **C288**, C415, C500, C582	None
FDP‐lysine	**K235**	N.A.	N.A.

*Note:* All these modifications were confirmed by nLC‐MALDI‐LIFT‐TOF/TOF MS/MS analysis of tryptic peptides after reaction at pH 8.0 and 37°C for 16 h. Underlined text indicates identifications from in‐gel digestion, while bold text indicates results from in‐solution digestion. Asterisks denote modifications by APAL itself (Schiff base, +55 Da), and not by APAL‐released ACRO. Numbering corresponds to the immature sequence in the UniProt database (Accession no. ALBU_BOVIN), with the mature protein shortened by 24 amino acids (representing the signal peptide and pro‐peptide sequences). N. A. stands for not available.

Ellmanʾs reagent was utilized to quantify thiol groups in YADH and BSA to compare differences before and after the modification reactions. The determined average amount of free thiols in YADH was 7.8 per molecule, reduced to 0.9 and 2.0 after the modifications with ACRO and FACRO, respectively. It was unchanged (8.2) after the reaction with APAL. For nBSA, the average numbers were 0.5 (no reaction), 0.6 (APAL), 1.1 (ACRO), and 1.0 (FACRO), which indicated no modification. Finally, for rBSA, the average numbers were 21.6 (no reaction), 1.1 (APAL), 1.0 (ACRO), and 0.5 (FACRO). As the predicted number of Cys is 35 per molecule of BSA, this quantification documented an incomplete reduction of the protein with DTT. On the other hand, it also indicated a large extent of modification of Cys upon the treatment.

**FIGURE 5:**
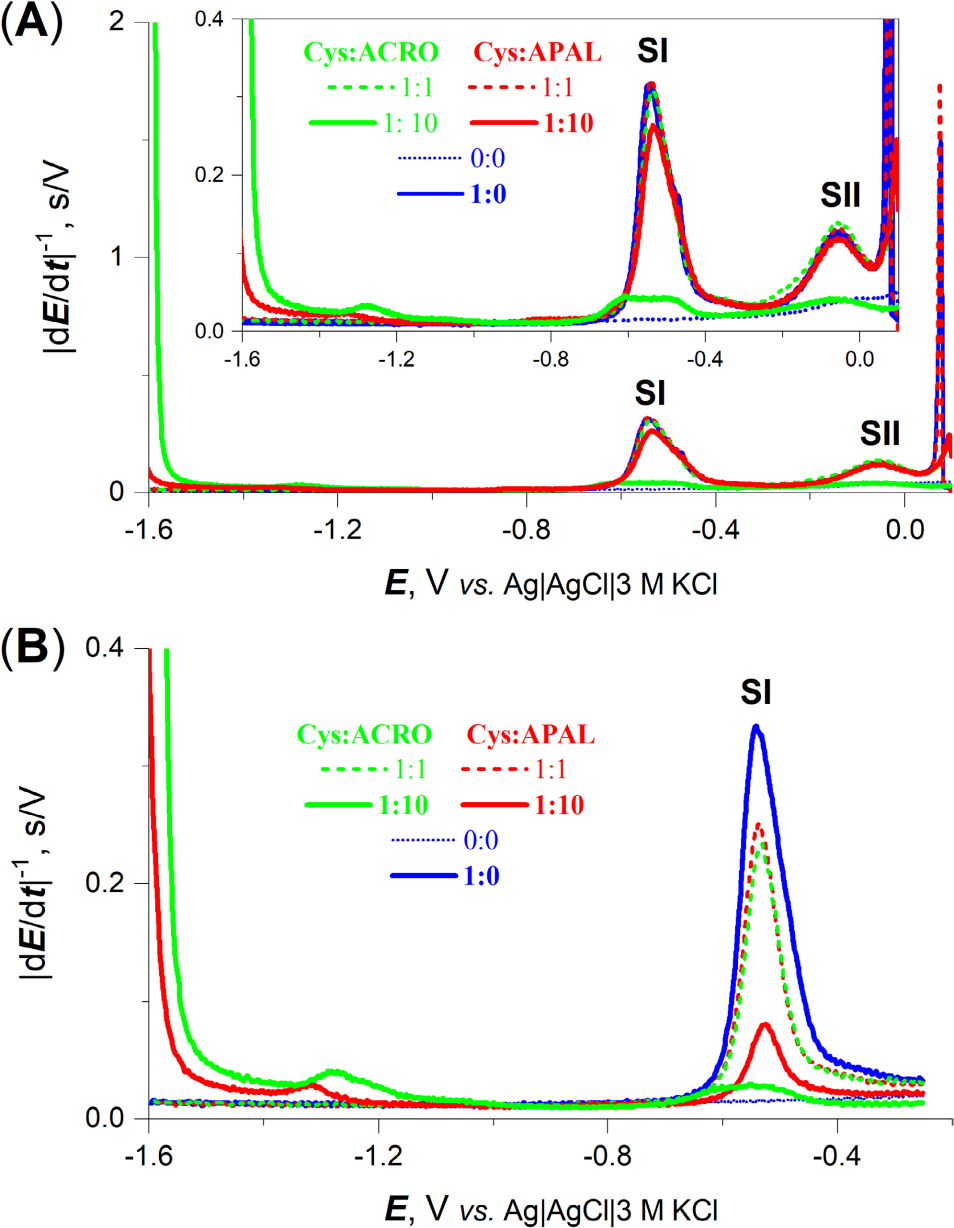
CPS peaks SI and SII of Cys treated with APAL or ACRO. The chronopotentiometric curves were measured at the indicated compound ratios and recorded after accumulation at a potential *E*_A_ of (A) 0.1 or (B) −0.25 V.

Figure 5 shows the results of CPS analyses of Cys and its mixtures with APAL or ACRO in different molar ratios. With ACRO, a significant decrease in both Cys peaks, SI and SII, at an electrode potential *E*_A_ of 0.1 V (Figure 5A) was observed, along with a decrease in peak SI at an *E*_A_ of −0.25 V (Figure 5B). However, for APAL, a significant decrease in the SI peak was only observed at an *E*_A_ of −0.25 V (Figure 5B). Peaks SII and SI reflect cysteine interactions with the electrode mercury. The peak SII reflects the reduction of a cysteine compound with Hg (II) to a cysteine compound with Hg(I), while the peak SI is attributed to the reduction of the cysteine compound with Hg(I) to Hg(0) and free Cys. According to the literature [26], Cys forms a compound with Hg (II) at an *E*_A_ of 0.1 V, while at an *E*_A_ of −0.25 V, it reacts with Hg (I). It has been shown [27] that, in addition to the cysteine SH‐group, its deprotonated NH_2_‐group binds to Hg (II). In the reaction with Hg(I), only the SH‐group of Cys is involved. These reactions are hindered when the Cys thiol group is modified by the reactive aldehydes studied.

## 4 Discussion

ACRO has been thoroughly characterized as a potent protein modifier, which is endogenously generated particularly from polyamine degradation, via aminoaldehydes [4]. The target amino acids (Cys, Lys, His, and Arg) and the type of modification reactions (formation of Michael or other adducts at Cys, Lys, His, and Arg; Schiff bases at Lys; FDP‐Lys; MP‐Lys) have long been known and analyzed using instrumental methods such as MS and NMR spectroscopy [28, 29, 30]. APAL, another product arising from polyamine degradation, has been shown to be a cytotoxic compound. It has been also postulated in the literature that it releases acrolein upon elimination of the amino group [4, 31]. However, protein modifications induced by APAL have not been studied so far. Therefore, our aim was to study these reactions using MALDI‐TOF MS, which was further supported by spectrophotometric and electrochemical analyses with Cys and its reacted forms.

Initial experiments were performed to analyze the reaction of APAL with the amino acids Cys (together with its derivatives/analogs NAcCys and CyA) and Lys. Based on the detected mass differences, free Cys as well as CyA probably form cyclic derivatives with APAL (+55 Da), containing a thiazolidine moiety, which is in accordance with the literature describing such a cyclization [32], while NAcCys forms a thiohemiacetal because of the presence of a blocked amino group (+73 Da). A Schiff base is obviously formed in the reaction with Lys (+55 Da). In parallel reactions with ACRO, Cys was modified to yield a Schiff base (+38 Da) and a Michael adduct (+56 Da). Lys was modified, yielding a Schiff base (also observed with CyA) and FDP‐Lys (+94). Finally, the obtained modifications induced by FACRO in Cys, Lys, and CyA were in accordance with the formation of Schiff bases (+104 Da), which is not possible in NAcCys. All these results confirm the reactivity of APAL, ACRO, and FACRO, and are consistent with theoretical predictions. The reaction of the thiol group in Cys with APAL and ACRO was further confirmed by electrochemical measurement of peak SI, which was reduced upon the addition of the aldehydes. This finding is in agreement with the previous study on cysteamine interactions with selected bioactive electrophiles [33].

Measurements with model peptides indicated that the N‐terminal amino group was sensitive to the reaction with APAL and ACRO, as it was found to be modified in all peptides (and in UBQ) except for insulin. Our observations were consistent with the previously reported release of ACRO from APAL via the elimination of ammonia. Typically, a Schiff base and a Michael adduct were found to occur from MS and MS/MS data, and the mass differences observed were the same with the use of APAL and ACRO. When employing ACRO, FDP‐ and MP‐like mass differences were also confirmed for the modified N‐termini. This could be elucidated by the presence of lower effective concentrations of the APAL‐derived ACRO in APAL‐containing reaction mixtures, which limited or precluded the reaction of two necessary ACRO moieties at the same site to form the cyclic derivatives. Other modification sites were targeted to Lys and Cys residues (the latter only with the reduced insulin) for APAL and ACRO, and additionally to Arg and His for ACRO. The most common modifications induced by APAL‐derived ACRO and detected in UBQ were MP‐Lys (55%–75% depending on the use of trypsin or Glu‐C, respectively, for the digestion), followed by Schiff bases (22%–25%), Michael adducts and FDP‐Lys (both 11%). Similarly, the highest proportion of MP‐Lys (50%–58%) was identified when using ACRO, the percentages of FDP‐Lys and Michael adducts were increased to 14%–16% and 20%–25%, respectively. The high amounts of the cyclic MP‐Lys and FDP‐Lys derivative could be due to the good accessibility of specific Lys residues. K48, K11 and K63 are known to be the most common Lys residues in ubiquitin linkages. They are present in surface loops. On the other hand K6, K27, K29, and K33 are less accessible and thus less available for a chemical reaction [34]. All possible modification types including the cyclic derivatives were only observed at K48. This is not surprising, as K48 is the most common Lys residue for polyubiquitin chain formation [35].

The two larger proteins used in this study, YADH and BSA, contain numerous Cys and Lys residues in their amino acid sequences (UniProt access. nos. ADH1_YEAST and ALBU_BOVIN, respectively). The corresponding counts for the mature protein chains are 8 Cys and 24 Lys for YADH, and 35 Cys and 59 Lys for BSA. Except for a single residue (C34), the cysteines in BSA participate in the formation of disulfide bonds. On the other hand, disulfides are absent in YADH. Also, there are numerous His and Arg residues present, which could be the target sites for modifications. The preferred target sites of APAL, ACRO, and FACRO in YADH were Lys residues, leading to the formation of Schiff bases. The lysines K28, K30, K81, K224, K226, K332, and K335 were modified by all the aldehyde compounds studied, indicating their susceptibility to this type of chemical reaction. Only C278 was confirmed to be a thiol‐containing target, despite spectrophotometric quantification revealing a higher level of cysteine modification. However, the data‐dependent precursor fragmentation in our nLC‐MALDI system obviously did not capture all the peptides with cysteine modifications. A mutual comparison shows that the same peptides are modified after the reactions with APAL and ACRO (*m/z* 1645, 1912, 2057), consistent with the expected formation of ACRO via APAL decomposition.

For BSA, native and reduced, not only lysines were modified, but also cysteines. The presence of modified Cys residues in nBSA probably originated from the use of reducing agents (2‐mercaptoethanol in Laemmli sample buffer, and DTT prior to alkylating samples to be digested and analyzed by MS), which caused an exposure of the released thiols to unreacted APAL‐derived ACRO or ACRO, which was in excess in the reduced protein sample. On the other hand, this was no obstacle to obtaining the desired comparative data.

**FIGURE 6:**
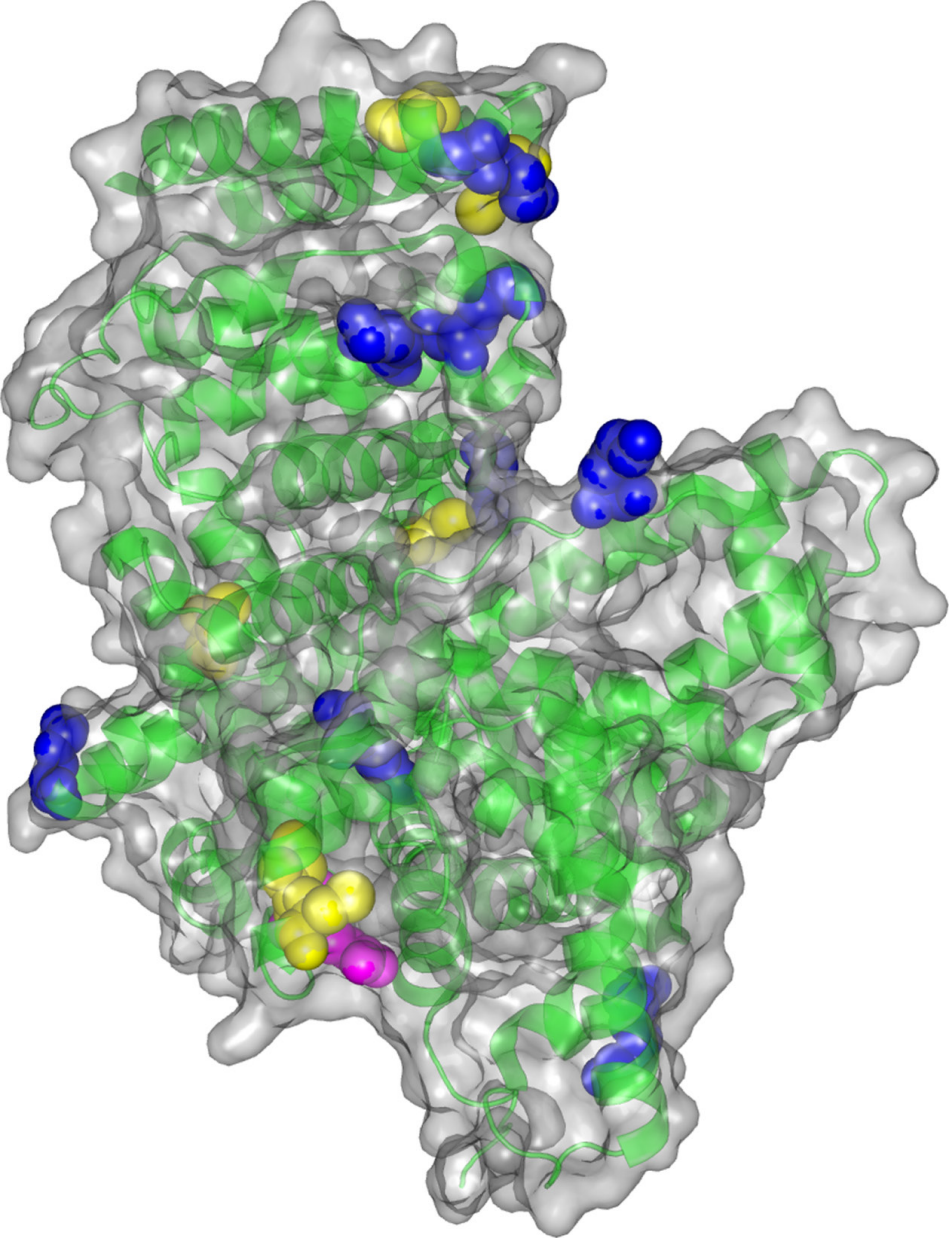
Modifications sites of APAL and APAL‐derived ACRO in BSA as deduced from MS/MS spectra. As can be seen, such sites are exposed at the surface. The target Cys residues (in rBSA) are highlighted as yellow spheres, Lys residues are in blue, and one relevant Arg residue is in magenta. The surface is in gray and green color depicts secondary structures. This visualization was created in PyMOL 1.3r2 (https://pymol.org) using the 3‐D structure obtained as a PDB formatted file downloaded from the PDB database (https://www.rcsb.org/; Accession No. 4F5S).

The majority of Cys residues from the reduced disulfides were finally found to be alkylated by iodoacetamide. The spectrophotometric quantification measurements confirmed the presence of only a single reduced Cys in nBSA, and also that APAL/ACRO modified many Cys residues in rBSA. The target sites of APAL and ACRO again largely overlapped. The following residues in BSA were confirmed to be modified by both APAL and ACRO: (1) Lys residues: K36, K140, K544, K547, K587; (2) Cys residues: C125, C288, C500, C582, C590; (3) other residues: R122. The presence of an ACRO Michael adduct at arginine is not surprising, as it has been reported [28]. Figure 6 shows the surface location of residues in BSA modified by APAL for illustration. Modifications of serum albumin by ACRO have been known from previous experiments. Amino acid analyses after the total hydrolysis of human serum albumin (HSA) modified at different concentrations of the aldehyde found Lys and His residues to be primary target sites (in a non‐reduced protein with disulfides). There was a proportional increase in the amount of adducts formed with increasing concentrations of ACRO [36]. Electrospray ionization mass spectrometry (ESI‐MS) was employed to study modifications induced by ACRO or 4‐hydroxynonenal in HSA and actin [37, 38, 39], which again targeted specific Lys and His residues, and also the free thiol of Cys34, yielding covalent adducts. This has been summarized in a recent review [40]. MALDI‐TOF MS was chosen due to its high throughput, tolerance to salts and buffer components, and its suitability for profiling peptide mixtures with minimal sample preparation [41]. While ESI‐MS, particularly in LC–MS/MS formats, offers superior sensitivity and rich fragmentation‐based structural elucidation, MALDI‐TOF MS provided adequate resolution and mass accuracy for detecting major modification products such as Schiff bases, Michael adducts, and cyclic adducts, and allowed rapid screening across multiple model peptides and proteins. However, we acknowledge that MALDI‐TOF MS is less effective in detecting low‐abundance adducts or labile intermediates compared to ESI‐MS. For the purposes of this study, namely the detection and comparative profiling of predominant acrolein‐induced modifications in model peptides and proteins, MALDI‐TOF MS proved to be a suitable and efficient analytical choice. Although the aldehyde concentrations used in this study exceed physiological levels, they enabled efficient detection and characterization of modification types and sites by MALDI‐TOF MS. These in vitro models provide mechanistic insight into residue susceptibility and reaction pathways. The predominance of acrolein‐derived adducts over APAL‐Schiff bases suggests that APAL exerts its effects mainly through conversion to acrolein, which forms biologically relevant modifications. While direct in vivo extrapolation is limited, the identified adducts support their potential as biomarkers of reactive carbonyl stress.

In conclusion, our study for the first time shows that ACRO and APAL modify peptides and proteins in a similar way, targeting overlapping residues and yielding largely the same modification products, including bound ACRO moieties such as Michael adducts and Schiff bases. Genuine APAL‐containing modifications (Schiff bases with Lys, + 55 Da) were rare. Compared to the effect of ACRO itself, the APAL‐derived ACRO only rarely and weakly forms FDP‐Lys, and MP‐Lys also occurs less frequently, which probably stems from its lower effective concentration in parallel comparative experiments, as two aldehyde moieties are necessary at the same site [29]. While high aldehyde concentrations were used to facilitate analytical detection, the resulting adduct profiles provide a useful model for understanding residue susceptibility and reaction mechanisms. The predominance of ACRO‐derived modifications and the negligible presence of stable APAL‐containing adducts suggest that APAL may act primarily as a reactive precursor releasing ACRO rather than a direct modifier of proteins in vivo.

## Conflicts of Interest

The authors declare no conflicts of interest.

## Data Availability

The data that support the findings of this study are available from the corresponding author upon reasonable request.
